# Fatty Liver Index for Predicting Nonalcoholic Fatty Liver Disease in an Asymptomatic Korean Population

**DOI:** 10.3390/diagnostics11122233

**Published:** 2021-11-29

**Authors:** Eun-Ju Cho, Gu-Cheol Jung, Min-Sun Kwak, Jong-In Yang, Jeong-Yoon Yim, Su-Jong Yu, Goh-Eun Chung

**Affiliations:** 1 Department of Internal Medicine and Liver Research Institute, Seoul National University College of Medicine, Seoul 03080, Korea; creatioex@gmail.com (E.-J.C.); sujongyu@gmail.com (S.-J.Y.); 2 Healthcare Research Institute, Gangnam Healthcare Center, Seoul National University Hospital, Seoul 06236, Korea; jungkc123@naver.com; 3 Department of Internal Medicine and Healthcare Research Institute, Gangnam Healthcare Center, Seoul National University Hospital, Seoul 06236, Korea; kms39@snuh.org (M.-S.K.); drmirinae@snuh.org (J.-I.Y.); yjy@snuh.org (J.-Y.Y.)

**Keywords:** fatty liver index, predict, cutoff, steatosis

## Abstract

Nonalcoholic fatty liver disease (NAFLD) is increasing worldwide, highlighting the importance of early and accurate detection and the appropriate management of NAFLD. However, ultrasonography (US) is not included in many mass screening programs, and people have limited access to it. The aim of this study is to validate the fatty liver index (FLI) and investigate the optimal cutoff value for predicting NAFLD in an asymptomatic population. We conducted a retrospective cohort study in Korea. All subjects who underwent health checkup exams, including abdominal US, controlled attenuation parameter (CAP) and blood testing, were enrolled. Analyses of the area under the receiver operating characteristic curve (AUROC) were used to evaluate the diagnostic accuracy and to calculate the optimal FLI cutoff for US-NAFLD. Among the 4009 subjects (mean age 54.9 years, 83.5% male), the prevalence of US-diagnosed NAFLD and CAP-defined hepatic steatosis was 61.4% and 55.4%. The previously used cutoff of FLI = 60 showed poor performance in predicting US-diagnosed NAFLD, with an AUROC of 0.63 (0.62–0.64), and CAP-defined NAFLD, with an AUROC 0.63 (0.62–0.64). The optimal FLI cutoff values to discriminate fatty liver detected by US were 29 for the entire population, with an AUROC of 0.82 (0.81–0.84). The sex-specific values were 31 for males and 18 for females (sensitivity 72.8% and 73.4%; specificity 74.2% and 85.0%, respectively). The FLI cutoff for US-diagnosed NAFLD can be set as 29 for the entire Korean population. Considering the sex dimorphism in NAFLD, different cutoff values are suggested to predict US-diagnosed NAFLD. These results may be helpful in the accurate non-invasive diagnosis of NAFLD.

## 1. Introduction

Non-alcoholic fatty liver disease (NAFLD) is the most common cause of chronic liver disease worldwide, with a prevalence of 25% globally and 27% in Asia [1,2]. Although most NAFLD shows a benign clinical course, some patients progress to advanced liver disease, including cirrhosis and hepatocellular carcinoma [3]. Moreover, NAFLD is closely related to various extrahepatic metabolic conditions, including obesity, insulin resistance, type 2 diabetes and cardiovascular diseases [4]. Thus, early and accurate diagnosis and appropriate management of NAFLD are important.

Ultrasonography (US) is considered a first-line screening tool for the diagnosis of NAFLD in clinical practice guidelines [5,6]. As a new modality for detecting liver steatosis, the controlled attenuation parameter (CAP) during transient elastography using FibroScan^®^ is considered to be an accurate method for the detection and quantification of hepatic steatosis. CAP can be used for the early and noninvasive detection of NAFLD at the subclinical stage [7,8,9] and is considered a reference for diagnosing hepatic steatosis [10]. However, US and CAP are not included in many mass screening programs, such as the Korean National Health Insurance System; thus, the fatty liver index (FLI), a proxy marker of hepatic steatosis, has been used to measure NAFLD instead of US in many studies using claim data [11,12].

The FLI is calculated by an equation that accounts for waist circumference (WC), body mass index (BMI), triglycerides and gamma glutamyl transpeptidase (GGT). It was first proposed by Bedogni et al. to predict fatty livers. In an Italian population, an FLI < 30 was used to rule out hepatic steatosis, and an FLI ≥ 60 was used to diagnose NAFLD [13]. Huang et al. suggested that the FLI cutoff value of 30 for Chinese individuals had a high sensitivity and specificity [14]. A recent study reported that the optimal FLI cutoffs in females and males were 10 and 20, respectively, suggesting that a lower cutoff should be applied in Asian people compared to Western countries [15]. Similarly, Yang et al. reported lower FLI cutoff values of 35 for males and 20 for females in a Taiwanese population [16]. In the Korean population, an FLI ≥ 60 was previously validated to correspond to US-diagnosed fatty liver, and the optimal FLI cutoff was suggested to be 39.9 [17,18]. However, these results are limited because the cutoff value was not stratified by sex and the sample size was small. Thus, we investigated the optimal FLI cutoff values using US or CAP as the reference method in an asymptomatic health checkup population.

## 2. Methods

### 2.1. Study Population

We conducted a retrospective cohort study based on subjects who underwent health checkups at the Seoul National University Hospital Health Care System Gangnam Center between January 2018 and December 2019. This institution has several health checkup programs that specialize in specific organs. This study included subjects who underwent various liver-related exams on the same day, including abdominal US, FibroScan (Echosens, Paris, France) and laboratory tests. The subjects were mostly free of symptoms and underwent the examinations upon their own request or through employer-sponsored coverage.

A total of 5804 subjects were initially enrolled. For NAFLD, subjects who displayed any potential cause of chronic liver disease were excluded: 332 were positive for the hepatitis B virus, 51 were positive for the hepatitis C virus and 1314 had a significant alcohol intake (>20 g/day for males and >10 g/day for females) [6]. Additionally, 98 subjects with missing information were excluded. Finally, 4009 subjects were included for analysis.

### 2.2. Clinical Parameters and Biochemical Analysis

As previously described [19], standardized self-report questionnaires were used to collect data at the time of enrollment. BMI was calculated as weight (kg) divided by the square of the person’s height (m). Well-trained personnel measured the WC at the midpoint between the lower costal margin and the iliac crest. Systolic and diastolic blood pressures were measured twice on the same day. A systolic/diastolic blood pressure ≥ 140/90 mm Hg and/or previous use of antihypertensive medication were used to define hypertension. Fasting glucose levels ≥ 126 mg/dL and/or oral hypoglycemic agent or insulin treatment were defined as clinical presentations of diabetes mellitus.

After an overnight fast of ≥8 h, blood specimens were obtained from each participant. Laboratory tests included serum levels of fasting glucose, triglycerides, aspartate aminotransferase (AST), alanine transaminase (ALT), GGT and creatinine. The glomerular filtration rate (GFR) was calculated using the modification of diet in renal disease equation.

### 2.3. Noninvasive Markers for NAFLD Measurement

The FLI was calculated based on the following equation [13] and expressed as a value ranging from 0 to 100.

FLI = [(e^0.953×lntriglyceride+0.139×BMI+0.718×lnGGT+0.053×WC−15.745^)/(1 + e^0.953×lntriglyceride+0.139×BMI+0.718×lnGGT+0.053×WC−15.745^)] × 100

### 2.4. Measurement of Fatty Liver Using US and CAP

Experienced radiologists, who were kept blind from the clinical characteristics of the subjects, conducted hepatic ultrasonography (Acuson Sequoia 512; Siemens, Mountain View, CA, USA). Fatty liver was diagnosed based on characteristic ultrasonographic features consistent with a “bright liver”, evident contrast between hepatic and renal parenchyma, vessel blurring, focal sparing, and luminal narrowing of the hepatic veins [20].

The CAP was obtained by FibroScan using an M or XL probe (Echosens, Paris, France). The procedure was performed by an experienced investigator who was kept blind from the patients’ clinical information as previously described [21]. Briefly, the individual was positioned in dorsal decubitus with the right arm in a maximally abducted state, and the exam was performed on the right lobe through the intercostal spaces. Liver stiffness measurement (LSM) values were considered reliable if 10 valid measurements were obtained and the interquartile range/median of the measurements were <0.3, or when the LS median was <7.1 kPa. All of the patients with 10 valid measurements were included in the analysis. The CAP values measured simultaneously during LSM measurement were used. A CAP value of 248 dB/m or greater was used to define hepatic steatosis [9].

### 2.5. Statistical Analyses

The data are presented as the mean ± standard deviation for normally distributed, continuous variables and as proportions for categorical variables, unless otherwise indicated. Log transformations were performed for non-normally distributed variables. The comparison of baseline characteristics was conducted using independent t tests and analysis of variance for continuous variables and the chi-square test for categorical variables. Among variables with a *p* value < 0.05 in univariate analyses, those with clinical importance were subjected to multivariate analyses. A ROC curve was applied to establish the optimal FLI cutoff value to differentiate between individuals with or without fatty liver by US. The optimal cutoff was selected as the maximum Youden’s index (sensitivity + specificity-1) in the ROC curve analysis. The sensitivity (SN), specificity (SP), positive predictive value (PPV), negative predictive value (NPV), positive likelihood ratio (LR+), and negative likelihood ratio (LR–) were calculated. The statistical analyses were performed using SAS version 9.4 (SAS Institute, Cary, NC, USA) and R version 4.0.4 (R Project for Statistical Computing, Vienna, Austria, http://www.Rproject.org, accessed on 27 July 2021). A two-sided *p* value < 0.05 was considered statistically significant.

## 3. Results

### 3.1. Baseline Characteristics of the Study Population

The mean age of the study population was 54.9 years, and 83.5% were male. Among the 4009 subjects, the prevalence of US-diagnosed NAFLD was 61.4% and CAP-defined hepatic steatosis was 55.4%.

The baseline characteristics of the subjects stratified by sex according to the presence of NAFLD are shown in Table 1. In both males and females, individuals with NAFLD had higher BMI and WC values. Serum AST, ALT, GGT and triglyceride levels were significantly higher in subjects with NAFLD (*p* < 0.001). The prevalence of diabetes was also higher in individuals with NAFLD than in those without NAFLD. These trends were similar for both ultrasonography-diagnosed and CAP-defined NAFLD.

### 3.2. Validation of FLI for Predicting NAFLD

First, we validated previously known FLI cutoff values for ruling out and detecting NAFLD by comparing their AUROCs. Measures of discriminatory accuracy are provided in Table 2. An FLI < 30 to rule out US-diagnosed NAFLD showed a sensitivity of 71.4% and NPV of 63.0% with an area under the receiver operating characteristic curve (AUROC) of 0.74 (0.73–0.76). An FLI ≥ 60 to predict US-diagnosed NAFLD showed a specificity of 95.7% and a PPV of 91.6%, with an AUROC of 0.63 (0.62–0.64). In addition, an FLI < 30 to rule out CAP-defined NAFLD had a sensitivity of 71.5% and a NPV of −66.7%, with an AUROC of 0.71 (0.70–0.73). An FLI ≥ 60 to predict CAP-defined NAFLD had a specificity of 94.7% and a PPV of 87.9%, with an AUROC 0.63 (0.62–0.64).

### 3.3. Optimal FLI Cutoff Values for Predicting NAFLD

We evaluated the optimal FLI cutoff values for predicting US-diagnosed NAFLD. A cutoff value of FLI ≥ 29 (sensitivity 73.4%, specificity 76.1%) predicted US-diagnosed NAFLD with good accuracy, with an AUC of 0.82 (0.81–0.84) (Table 3).

**Figure 1:**
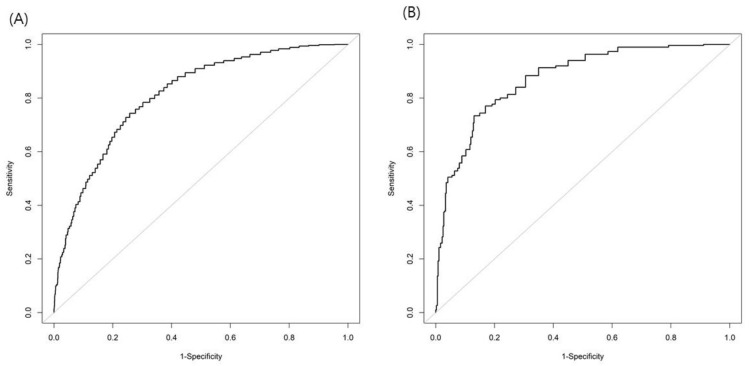
Area under the receiver operating characteristics curve of the fatty liver index for predicting nonalcoholic fatty liver disease in males (**A**) and females (**B**).

Because the prevalence of NAFLD is different according to sex, the FLI cutoff analysis was conducted separately for males and females. A cutoff value of FLI ≥ 31 (sensitivity 72.8%, specificity 74.2%) for males and FLI ≥ 18 (sensitivity 73.4%, specificity 85.0%) for females predicted US-diagnosed NAFLD (Table 3). Figure 1 illustrates the FLI AUROCs for the prediction of US-diagnosed NAFLD in males and females.

## 4. Discussion

In this study, we validated the FLI in an asymptomatic Korean population. Considering that AUCs between 0.8–0.9 are usually regarded as good values and those between 0.7–0.8 as fair values, the previously used cutoff of FLI = 60 performed poorly in predicting NAFLD in the Korean population. In our study, to predict NAFLD by US diagnosis in the Korean population, we proposed optimal FLI cutoff values of 29 for the entire population, 31 for males and 18 for females. These results may be helpful to identify asymptomatic individuals who should undergo abdominal US screening for NAFLD diagnosis.

In Western populations, the previously validated cutoff values have shown good accuracy, with AUROCs of 0.81–0.84 [13,22] and an FLI < 30 rules out hepatic steatosis (sensitivity = 87%) and an FLI ≥ 60 predicts the condition (specificity = 86%). When the same cutoff (FLI ≥ 60) was applied to an Asian population, although the accuracy was similarly good (AUC 0.87), the Youden index decreased to 23–27% [18]. In the current study, an FLI ≥ 60 to predict US-diagnosed NAFLD showed a similar performance (specificity = 95.7%); however, an FLI < 30 to rule out NAFLD showed relatively lower performance (sensitivity = 71.4%). These results suggest that different cutoff values are needed for the Asian population. In an Asian population, the optimal FLI cutoff has been suggested to be 30 for middle-aged to elderly Chinese individuals, presenting a maximum Youden’s index of 0.51 and achieving a high sensitivity of 79.9% and a specificity of 71.5% [14]. In addition, Kim et al. suggested an FLI cutoff of 36.9, with a sensitivity of 77.4% and specificity 69.8% in a Korean population [17].

Because NAFLD is a sexually dimorphic disease with respect to epidemiological and clinical features [23], a sex-specific approach is required to establish optimal FLI cutoff values. A study performed in Taiwan that stratified by sex suggested optimal values of FLI ≥ 35 (specificity 79.8%, LR+: 3.12) for males and ≥20 (specificity 86.8%, LR+: 4.43) for females to predict US-diagnosed fatty liver [16]. Chen et al. suggested FLI cutoff values of 20 for males and 10 for females, with sensitivities of 80.3% and 76.1% and specificities of 66.9% and 65.5%, respectively [15]. Consistent with previous results, we presented data classified according to sex, and identified an FLI ≥ 31 (sensitivity 72.8%, specificity 74.2%) for males and an FLI ≥ 18 (sensitivity 73.4%, specificity 85.0%) for females to predict US-diagnosed NAFLD.

The FLI cutoff values in our study were lower for both sexes compared to those proposed by Bedogni et al. [13]. A possible reason for this is that, according to the Joint Interim Statement of the International Diabetes Federation Task Force on Epidemiology and Prevention, the metabolic syndrome criteria cutoff values for WC, which are a major component of the FLI, are set lower for the Asian population (90 cm for males and 80 cm for females) [24]. Thus, the low WC values likely led to the lower cutoff values of FLI in the current study, especially for females. Additionally, the Asia–Pacific population criteria for obesity are lower than those for Western populations (such as a BMI ≥ 25 kg/m^2^ [25]), which might have influenced the lower FLI cutoff values in the current study. Further studies are needed to validate these results.

In this study, we used CAP as an alternative method for diagnosing hepatic steatosis. The AUROC of FLI < 30 to rule out CAP-defined NAFLD was 0.71 and that of FLI ≥ 60 to predict NAFLD was 0.63—similar to US as a reference method. Although CAP measurement is a good noninvasive biomarker of fatty liver [9], further studies are need using CAP as a reference method for diagnosing NAFLD.

The present study has some limitations. First, we were unable to obtain liver histological samples, which is the gold standard for diagnosing NAFLD. Although US may produce false negative results when fatty infiltration of the liver is less than 20–30% [26,27], representing inter- and intra-observer diagnostic variability, ethical restrictions prohibit the application of invasive tests in apparently healthy populations. Thus, radiographic techniques such as US or magnetic resonance imaging are used as a first-line modality to diagnose NAFLD in clinical practice guidelines [5]. Regarding CAP, the ideal cutoff CAP values for detecting and grading steatosis have not yet been established [28,29]. However, the threshold used to diagnose hepatic steatosis in this study is similar to those in previous studies [10,30]. Second, since not all people who undergo health checkups at our institution receive a FibroScan exam, there may be selection bias, because we included only the subset of the screened individuals who underwent the related exams. Furthermore, the cutoff values established in this study have not been externally validated in other populations. Therefore, the results of our study should be interpreted carefully, and further studies are needed to validate our results. Finally, we could not evaluate the severity of US-diagnosed NAFLD or the stage of fibrosis in this study.

In conclusion, the FLI cutoff for US-diagnosed NAFLD can be set as 29 for the entire Korean population. Considering the sex dimorphism in NAFLD, different cutoff values are suggested—31 for males and 18 for females—to predict US-diagnosed NAFLD. These results may be helpful in the accurate non-invasive diagnosis of NAFLD.

## Figures and Tables

**Table 1:** Baseline characteristics of the study population stratified by sex.

Male	No NAFLD (N = 1187)	US-NAFLD (N = 2162)	*p*-Value	No NAFLD (N = 1430)	CAP-NAFLD (N = 1918)	*p*-Value
Age (years)	55.5 ± 11.6	54.4 ± 9.7	0.008	55.2 ± 10.9	54.5 ± 10.1	0.091
Diabetes, n (%)	111 (9.4)	385 (17.8)	<0.001	142 (9.9)	354 (18.5)	<0.001
Hypertension, n (%)	247 (20.8)	488 (20.7)	0.958	301 (21.0)	394 (20.5)	0.721
Systolic blood pressure (mmHg)	120.9 ± 14.0	120.5 ± 13.8	0.432	120.7 ± 13.9	120.5 ± 13.9	0.772
Diastolic blood pressure (mmHg)	79.2 ± 10.2	79.8 ± 10.4	0.188	79.3 ± 10.2	79.8 ± 10.4	0.172
BMI (kg/m^2^)	23.3 ± 2.5	25.9 ± 3.0	<0.001	23.4 ± 2.4	26.1 ± 3.0	<0.001
WC (cm)	85.8 ± 7.3	92.8 ± 7.8	<0.001	86.0 ± 6.9	93.6 ± 7.8	<0.001
AST (IU/L)	26.2 ± 11.5	28.6 ± 13.7	<0.001	25.9 ± 10.3	29.1 ± 14.6	<0.001
ALT (IU/L)	27.7 ± 20.0	33.3 ± 23.3	<0.001	27.7 ± 17.9	34.0 ± 24.8	<0.001
GGT (IU/L)	30.0 ± 27.5	41.4 ± 33.1	<0.001	31.6 ± 28.8	41.4 ± 33.1	<0.001
Triglyceride (mg/dL) ^+^	83 (62,114)	128 (92,177)	<0.001	91 (66,126)	127 (90,179)	<0.001
GFR, mL/min/1.73 m^2^	84.7 ± 15.2	85.8 ± 14.2	0.034	84.6 ± 15.2	86.0 ± 14.2	0.006
CAP dB/m	224 (200,248)	278 (247,309)	<0.001	218 (198,233)	289 (268,315)	<0.001
NAFLD detected by USG, n (%)				544 (38.0)	1617 (84.3)	<0.001
Female	No NAFLD (N = 360)	US-NAFLD (N = 301)	*p*-value	No NAFLD (N = 357)	CAP-NAFLD (N = 304)	*p*-value
Age (years)	53.8 ± 10.6	57.6 ± 9.1	<0.001	53.3± 10.3	58.1 ± 9.2	<0.001
Diabetes, n (%)	12 (3.3)	57 (18.9)	<0.001	18 (5.0)	51 (16.8)	<0.001
Hypertension, n (%)	78 (21.7)	72 (23.9)	0.491	85 (23.8)	65 (21.4)	0.458
Systolic blood pressure (mmHg)	120.1 ± 14.4	121.6 ± 14.7	0.175	121.6 ± 14.8	119.9 ± 14.2	0.137
Diastolic blood pressure (mmHg)	80.0 ± 9.5	81.0 ± 10.2	0.342	80.7 ± 9.4	80.0 ± 10.3	0.424
BMI (kg/m^2^)	22.3 ± 2.7	24.9 ± 3.4	<0.001	21.4 ± 2.8	24.8 ± 3.5	<0.001
WC (cm)	79.1 ± 7.6	88.1 ± 8.4	<0.001	79.3 ± 7.9	87.8 ± 8.4	<0.001
AST (IU/L)	24.5 ± 11.2	26.7 ± 13.6	0.023	24.3 ± 11.3	27.0 ± 13.3	0.006
ALT (IU/L)	27.5 ± 23.3	29.3 ± 18.0	0.259	26.7 ± 23.4	30.2 ± 17.8	0.029
GGT (IU/L)	19.3 ± 12.0	30.5 ± 36.4	<0.001	19.7 ± 14.6	29.8 ± 35.3	<0.001
Triglyceride (mg/dL) ^+^	67 (49, 88.5)	112 (78,152)	<0.001	66 (49, 89)	110 (77,148)	<0.001
GFR, mL/min/1.73 m^2^	84.1 ± 14.9	86.6 ± 14.5	0.033	84.4 ± 15.4	86.3 ± 13.9	0.090
CAP dB/m	213 (189,240)	283 (256,319)	<0.001	209 (185,229)	290 (268,319)	<0.001
NAFLD detected by USG, n (%)				62 (17.4)	238 (78.6)	<0.001

Data are shown as the mean ± SD. ^+^ median (interquartile range). NAFLD, nonalcoholic fatty liver disease; US, ultrasonography; CAP, controlled attenuation parameter; BMI, body mass index; WC, waist circumference; AST, aspartate aminotransferase; ALT, alanine aminotransferase; GGT, gamma glutamyl transpeptidase; GFR, glomerular filtration rate.

**Table 2:** Comparison of AUCs of fatty liver index for predicting nonalcoholic fatty liver disease.

	Cut Point	Sensitivity	Specificity	PPV	NPV	AUC (95% C.I.)	pLR	nLR
US	30	71.4%	77.4%	83.4%	63.0%	0.74 (0.73–0.76)	3.2	0.4
	60	29.3%	95.7%	91.6%	46.0%	0.63 (0.62–0.64)	6.9	0.7
CAP	30	71.5%	71.0%	75.4%	66.7%	0.71 (0.70–0.73)	2.5	0.4
	60	31.2%	94.7%	87.9%	52.5%	0.63 (0.62–0.64)	5.9	0.7

AUC, area under the receiver operating characteristic curve; PPV, positive predictive value; NPV, negative predictive value; C.I., confidence interval; pLR, positive likelihood ratio; nLR, negative likelihood ratio; US, ultrasonography; CAP, controlled attenuation parameter.

**Table 3:** Optimal cut-off levels of fatty liver index for predicting ultrasonography-diagnosed NAFLD.

	Cut Points	AUC (95% C.I.)	Youden’s J Index	Sensitivity	Specificity	PPV	NPV
Total	29	0.82 (0.81–0.84)	0.495	73.4%	76.1%	83.0%	64.2%
Male	31	0.74 (0.72–0.75)	0.470	72.8%	74.2%	83.7%	60.0%
Female	18	0.79 (0.76–0.82)	0.584	73.4%	85.0%	80.4%	79.3%

NAFLD, nonalcoholic fatty liver disease; AUC, area under the receiver operating characteristic curve; PPV, positive predictive value; NPV, negative predictive value; C.I., confidence interval.

## Data Availability

The data presented in this study are available on request from the corresponding author.
